# The Relationship between *CmADHs* and the Diversity of Volatile Organic Compounds of Three Aroma Types of Melon (*Cucumis melo*)

**DOI:** 10.3389/fphys.2016.00254

**Published:** 2016-06-28

**Authors:** Hao Chen, Songxiao Cao, Yazhong Jin, Yufan Tang, Hongyan Qi

**Affiliations:** ^1^Key Laboratory of Protected Horticulture of Ministry of Education and Liaoning Province, College of Horticulture, Shenyang Agricultural UniversityShenyang, China; ^2^Department of Horticulture, College of Agriculture, Heilongjiang Bayi Agricultural UniversityDaqing, China

**Keywords:** volatiles organic compounds, alcohol dehydrogenase, oriental melon, fruit ripening, gene expression

## Abstract

Alcohol dehydrogenase (ADH) plays an important role in aroma volatile compounds synthesis of plants. In this paper, we tried to explore the relationship between *CmADHs* and the volatile organic compounds (VOCs) in oriental melon. Three different aroma types of melon were used as materials. The principle component analysis of three types of melon fruit was conducted. We also measured the *CmADHs* expression level and enzymatic activities of ADH and alcohol acyl-transferase (AAT) on different stages of fruit ripening. An incubation experiment was carried out to investigate the effect of substrates and inhibitor (4-MP, 4-methylpyrazole) on *CmADHs* expression, ADH activity, and the main compounds of oriental melon. The results illustrated that ethyl acetate, hexyl acetate (E,Z)-3,6-nonadien-1-ol and 2-ethyl-2hexen-1-ol were the four principal volatile compounds of these three types of melon. AAT activity was increasing with fruit ripening, and the AAT activity in CH were the highest, whereas ADH activity peaked on 32 DAP, 2 days before maturation, and the ADH activity in CB and CG were higher than that in CH. The expression pattern of 11 *CmADH* genes from 24 to 36 day after pollination (DAP) was found to vary in three melon varieties. *CmADH4* was only expressed in CG and the expression levels of *CmADH3 and CmADH12* in CH and CB were much higher than that in CG, and they both peaked 2 days before fruit ripening. Ethanol and 4-MP decreased the reductase activity of ADH, the expression of most *CmADHs* and ethyl acetate or hexyl acetate contents of CB, except for 0.1 mM 4-MP, while aldehyde improved the two acetate ester contents. In addition, we found a positive correlation between the expression of *CmADH3* and *CmADH12* and the key volatile compound of CB. The relationship between *CmADHs* and VOCs synthesis of oriental melon was discussed.

## Introduction

Oriental melon (*Cucumis melo* var. *makuwa* makino) is a species of thin-pericarp melon, and it has extensive cultivated varieties and the largest plantation in china. The oriental melon has a sweet and crisp taste, juicy flesh and an edible rind, especially intense volatile aromas compound that is one of the most attractive qualities (Liu et al., 2012). Most volatile aroma compounds, as a sign of fruit maturity, are produced and released during the maturation period (Visai and Vanoli, 1997; Goff and Klee, 2006). To date, more than 2000 types of volatile compounds have been detected in various plants, including melons, apples, strawberries, pears, tomatoes, and bananas (Dixon and Hewett, 2000; Maul et al., 2000; Urruty et al., 2002; Li et al., 2014, 2016). In different melon varieties, ~240 volatile compounds have been found, including volatile alcohols, aldehydes, terpene, especially abundant esters (Kourkoutas et al., 2006; Khanom and Ueda, 2008; Obando-Ulloa et al., 2010). Specifically, the contents of aromatic compounds vary drastically according to the melon variety. In climacteric melon varieties, volatile esters are prominent, together with short-chain alcohols, aldehydes and terpenes, while non-aromatic varieties often have much lower levels of total volatiles, lacking the volatile esters (Gonda et al., 2010). Tang also found that ester, especially straight-chain esters were important VOCs in oriental melon (Tang et al., 2015). As the most abundant aroma in climacteric melon, esters are mainly produced from two ways, namely the amino acid way, producing the branched-chain esters and the lipoxygenase (LOX) way synthesizing the straight-chain esters (Zhang et al., 2014; Tang et al., 2015).

The lipoxygenase (LOX) pathway may be the most critical way for aroma foundation because of the high straight-chain esters content of oriental melon. The LOX way consist of four enzymes, including LOX, HPL (Hydroperoxide lyase), ADH (Alcohol dehydrogenase, EC1.1.1.1), and AAT (Alcohol acetyltransferase). As the last two steps in the foundation of volatile esters, some ADH and AAT have been extensively investigated, both in melons and in other plants. These steps involve alcohol dehydrogenase and alcoholacetyl transferase activities that convert volatile aldehydes to their respective alcohols and esters, and these activities are related to climactericity (Gonda et al., 2010).

The classic ADHs are Z-binding enzymes, relying on an NAD(P) co-factor to interconvert ethanol and acetaldehyde (and other short linear alcohol/aldehyde pairs). In petunia, *PhADH2* and *PhADH3* were involved in floral scent from the lipoxygenase pathway (Garabagi and Strommer, 2004). Previous reports also showed that *ADHs* were expressed in a developmentally-regulated manner, particularly during fruit ripening (Salas and Sánchez, 1998; Speirs et al., 2002; Lara et al., 2003; Manríquez et al., 2006). Over-expression of *LeADH2* in tomato led to increasing the level of alcohols, particularly Z-3-hexenol of the fruit (Salas and Sánchez, 1998). The specific down-regulation of *SlscADH1* in tomato fruit did not alter the aldehyde/alcohol balance of the volatiles compounds, but made higher concent of C5 and C6 volatile compounds from the lipoxygenase pathway (Moummou et al., 2012). However, there were few reports on ADHs, participating in aroma synthesis, in oriental melon which has the extensive cultivated varieties and the largest plantation in China.

As our previous works, 12 *CmADH* genes (*CmADH1-12*) have been identified in the melon genome (http://melonomics.net/) and bioinformatics analyzed. We have also investigated the response of 12 *CmADHs* to ethylene in oriental melon (Jin et al., 2016), but the function of most members were far from clear, except for *CmADH1* and *CmADH2* in Countloup melon. The key *CmADH* gene participating in the accumulation of various volatile organic compounds (VOCs) in different aroma types of melon and the regulation of *CmADHs* family in the process of aroma foundation in oriental melon are still unknown. In this paper, to explore the potential *CmADH* genes participating in the key aroma compounds production, we analyzed the VOCs and investigated the activities and expression of ADH and AAT in ripening fruits of three different aroma types of melon. Simultaneously, a fruit disk incubation experiment was conducted to investigate the influence of substrates (ethanol and aldehyde) or inhibitor on ADH activity, *CmADHs* expression and VOCs productions in oriental melons.

## Materials and methods

### Plant materials

Three different aromatic oriental melon varieties were used, including strong- aromatic melon (*C. melo* var*. makuwa* Makino) cultivar “Cai Hong” (CH), less-aromatic melon (*C. melo* var*. makuwa* Makino) cultivar “Cui Bao” (CB), and non-aromatic melon “Cai Gua” (CG) which is called as snake melon (*C. melo L* var*. flexuosus Naud*) in China. They were grown in pots (volume of 25 L and soil: peat: compost = 1: 1: 1) in a greenhouse under standard cultural practices for fertilization and pesticide treatments at Shenyang Agricultural University(Shenyang, China) from March to June in 2014. Female flowers were pollinated with “Fengchanji 2” to increase the rate of fruit set, and tagged on the day of bloom. Melons were harvested on 24, 26, 28, 30, 32, 34, 36 days after pollination (DAP).

### Fruit firmness, soluble solids content (SSC) evaluation

The firmness of melon fruit was measured with a hardness tester (FHM-1, Takemura, Japan) according to the method of Tijskens (Tijskens et al., 2009). The soluble solids content of melon fresh was determined by a digital refractometer (DBR45, Huixia, Fujian, China) described by Liu (Liu et al., 2012). A CR-400/410 spectrophotometer (Konica Minolta, Japan) was used to detect the rind color of melons. Six readings were taken from equatorial zone of each fruit (Liu et al., 2012). The firmness and SSC experiment was performed in triplicate.

### ADH enzyme activity assay

Reductase and dehydrogenase activities of ADH were evaluated by CARY 100 scan ultraviolet (UV)/visible spectrophotometer (Varian, USA). The method was optimized on the foundation of Longhurst et al. (1990) and Manríquez et al. (2006). Approximately 3 g fresh melon was ground into powder in liquid nitrogen using mortar and pestle, then mixed with 6 ml pre-cooling extract buffer [4°C, 100 mM MES-Tris (pH 6.5), 2 mM DTT (dithiothreitol), 1% PVP (polyvinyl pyrrolidone) (m/v)]. The ground slurry was centrifugated at 15,000 g for 30 min at 4°C, and the supernatant was collected for ADH activity analyzing as crude enzyme. Reductase activity was measured in 1 ml total volume containing 200 μl crude protein, 5 mM aldehyde, 0.25 mM NADH, or NADPH and 50 mM sodium phosphate buffer (pH 5.8). Dehydrogenase activity was assayed in solution contained 5 mM ethanol, 0.25 mM NAD or NADP and glycine-NaOH buffer pH 9.4 in 1 ml. Reductase/dehydrogenase activitywas measured by the increase/decrease in absorbance at 340 nm due to change of NAD(P). The reaction was initiated by the addition of ethanol or aldehyde and the rate of absorbance change without ethanol or aldehyde was subtracted to give the substrate dependent rate.

### AAT enzyme activity

AAT activity was measured according to Shalit (Shalit et al., 2001). Total protein was extracted from 3 g melon fruit without peel and macerated with 6 ml 0.1 M sodium phosphate buffer (pH = 0.8) at 4°C. The supernatant was collected as the crude enzyme for AAT activity analyzing after the mixture was centrifuged at 16,000 g for 30 min at 4°C. The reaction system consisted of 2.5 ml 5 M Mgcl_2_, 50 μl 0.5 mM acetyl CoA, 50 μl 200 mM butanol and 0.6 ml crude enzyme. 150 μl 5,5-disulfide double nitro benzoic acid (DTNB) was added into the mixture after 15 min. The AAT activity was determined by the changes of A412 measured by spectrophotometer and each measurement was repeated three times.

### Protein content

Total proteins were quantified with modifications (BioRad Protein Assay Kit, Bio-Rad, USA) according to the method of coomassie brilliant blue G-250 described by Bradford (Bradford, 1976).

### Volatile organic compounds analysis

The VOCs of different melons were detected under the procedure of headspace (HP)-solid phase micro extraction (SPME)-gas chromatography-mass spectrometry (GC-MS), as Liu and Tang was used (Liu et al., 2012; Tang et al., 2015). About 100 g frozen melon flesh were thawed and squeezed into juice. 1-octanol (50 μl, 59.5 mg/l) were added into 10 ml juice samples as an internal standard. SPME needle was from Supelco (57347-U, Bellefonte, PA, USA), and GC-MS was from Thermo Scientific (Trace GC Ultra-ITQ 900, Waltham MA 02454). The GC system was equipped with a 30 m^*^0.25 mm^*^0.25 um thickness capillary column (Thermo TR-5 ms SQC, USA).

For incubation experiment, a 1 g aliquot of the melon powder was placed in a 10 ml glass vial containing 0.7 g of solid NaCl, 2 ml of a 20 % (w/v) NaCl solution (Gonda et al., 2010) and 10 μl of a 59.5 mg/l 1-octanol used as internal standard. Then, the sample was measured with the method mentioned above.

### Incubation experiments

Melon cubes (4 g) from CB mature fruit were put in sterile petri dish plates and 500 μl of a solution of 5 mM ethanol or 5 mM aldehyde and different concentrations of ADH inhibitor (4-methylpyrazole, 4-MP. 0.1, 1, and 5 mM) were applied on top of each cube, and distilled water was taken as control. The plate was covered and incubated overnight at room temperature. Then, each cube was frozen in liquid nitrogen and stored at −80°C (Gonda et al., 2010).

### Real-time quantitative (qPCR) analysis

The total RNA was isolated with TRIzol Reagent (Takara, Japan). DNase I (Promega, USA) was used to remove genomic DNA. cDNA template was obtained by reverse transcriptase(Invitrogen, Thermo fisher scientific, USA) with random primer. The PCR program parameters consisted of a preliminary step of 3 min at 95°C followed by 45 cycles at 95°C for 15 s and at 60°C for 30 s, finally, 68°C 30 s. The template cDNA was amplified in a 20 μl reaction (2xSYBR Green PCR Master Mix, Tiangen Biotech Co. Ltd. Beijing, China) on an ABI 7500 sequence detection system. All qPCR experiments were performed in triplicate with different cDNA template. The ADH/18s rRNA ration for samples were related to the ratio for CH in **Figure 5** and for CB in **Figure 8** which were set to 1, respectively. The 2^−ΔΔCt^ method was used to calculate relative genes expression of the *CmADH* genes produced by real time PCR.

### Statistical analysis

A principal component analysis (PCA) was employed to identify the key aroma compounds of the three aromatic melon varieties according to their VOCs by the SPSS 20.0. And significant analysis was conducted by a one-way ANOVA following Duncan's multiple range tests for experiment at a *p* < 0.05 level. The figures were produced by Origin 9.0.

## Results

### Firmness, soluble solids content (SSC), and rind color

**Figure 1:**
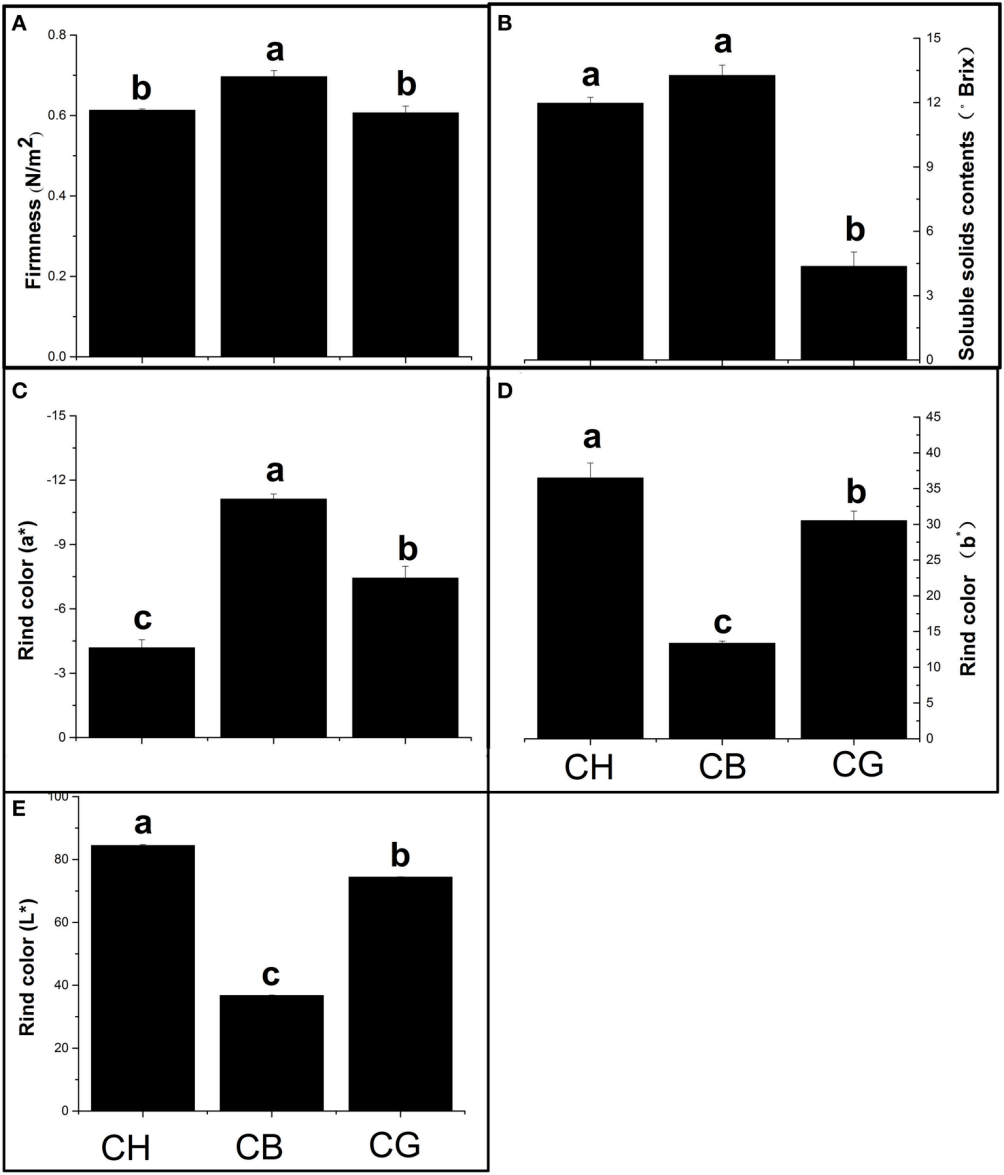
**Different physiological characteristics of three types of melon at their maturity. (A)** Firmness, **(B)** Soluble solids contents of melon flesh, **(C–E)** Pericarp color (a^*^ means the red/green ratio and b^*^ represents the yellow/blue ratio, L^*^ represents the brightness of rind). Duncan's multiple range tests have been performed with different letters above the columns represent significant differences (*P* < 0.05) between different types of melon.

In order to determine the maturation period, SSCs of the three types of melon was chosen for the signal of fruit maturation (Tang et al., 2015). We chose DAP34 as the maturation period of three melon, due to the directly relationship between SSCs and fruit development of melons. The SSC of three types of melon nearly reached the highest concentration at the same time at 34 DAP (Figure S1). The firmness of CH and CG were similar and lower than that of “CB” (Figure 1A). Both CH and CB fruit had higher SSCs than CG (Figure 1B). In terms of rind color, CH and CG were brighter or yellow, CB were dark green (Figures 1C–E; Figure S2). Moreover, CH, CB, and CG fruits also exhibited various morphological and physical characteristics, implying the ripening of different type melons.

### Volatile organic compounds of three types of melon

We had detected 49 VOCs, including esters, alcohols, acids, and other aroma in three types melons (Table S1). Esters were the most abundant volatiles in CH and CB (~207.83 μg.g^−1^FW and 127.16 μg.g^−1^FW, respectively). On the other hand, alcohol contributed the aroma of CG. We also found that the content of esters or total aroma accumulated in CH was nearly twice of those in CB, although esters was the main compounds in both of them (Table 1).

**Table 1:** **Total and different classes of volatile compounds and their concentrations in different aromatic melon types**.

**Volatile compounds**	**Different types of melon**		
**(μg.g^−1^FW)**	**CH**	**CB**	**CG**
Total esters	207.83 ± 17.21^a^	127.16 ± 16.75^b^	5.29 ± 1.82^c^
Total alcohols	30.24 ± 2.48^b^	23.68 ± 1.87^b^	136.85 ± 4.85^a^
Total acids	15.73 ± 5.28^a^	8.14 ± 2.62^b^	9.31 ± 3.97^b^
Others	54.15 ± 20.01^a^	10.07 ± 1.32^b^	6.14 ± 1.21^b^

*Duncan's multiple range tests were performed, and different letters represent significant differences (P < 0.05) between different types of melon*.

**Figure 2:**
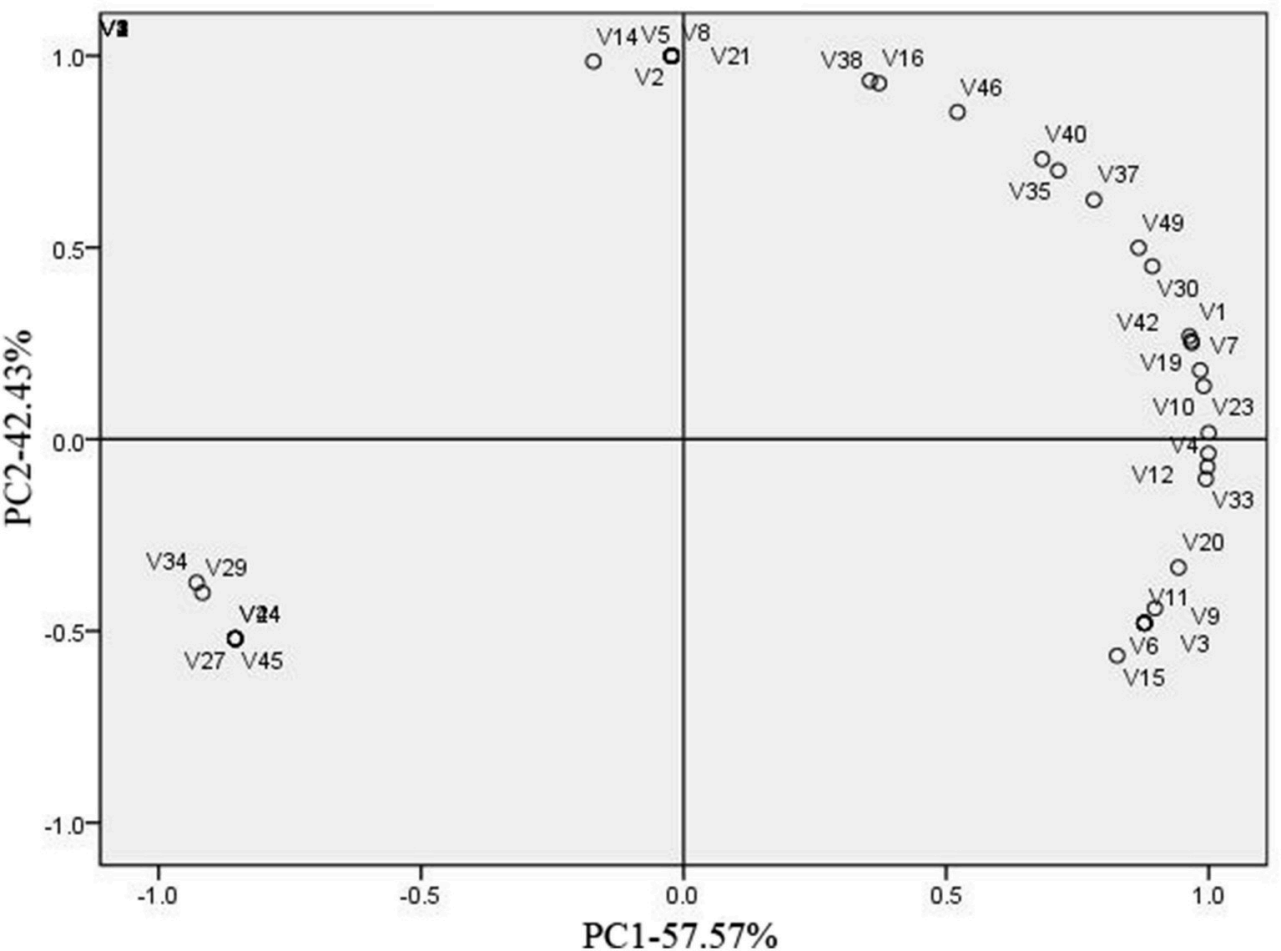
**Principal component analysis (PCA) of aroma volatiles identified in three types of melon at mature period**. Loading plots of the two main PCA of the aroma volatiles identified in three types of melon at mature period. One hundred percent of the variability in the volatile compounds in the melon cultivars could be explained by two principal PCs. PC1 explained 57.57% of the variability, while PC2 explained 42.43% of the variability. Each sample consisted of three replicates. Codes were corresponding to the volatile compounds number in Table S1.

**Figure 3:**
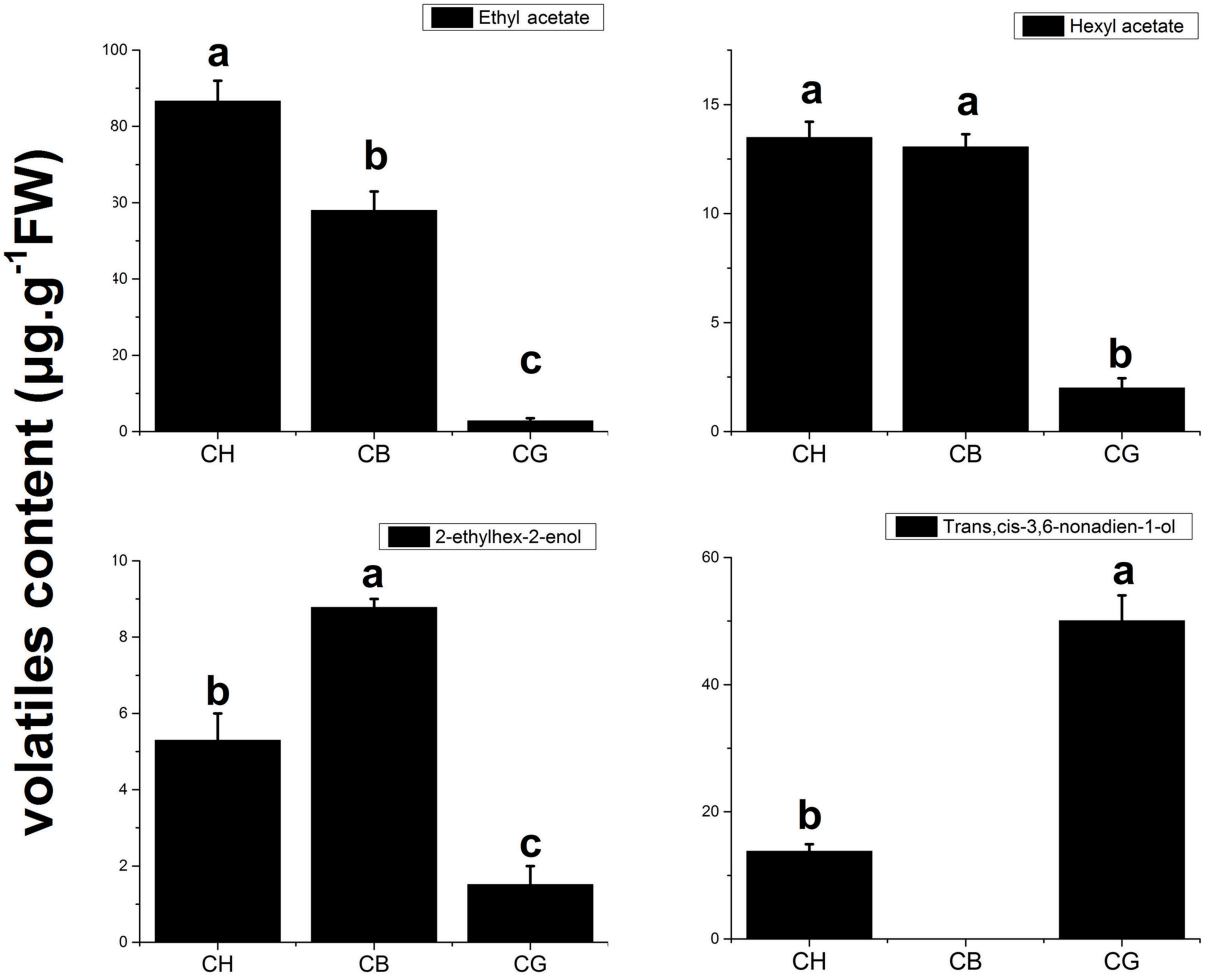
**Four principal volatile compounds of three types of melon at mature period**. All of the data for volatile compounds are means ± SE value of three replicates.

To further distinguish the variety of aroma in three types of melon, PCA of aroma volatiles identified in three types of melon at mature period was conducted (Figure 2). It was clearly that CG was separated from the others in account of V29 [(E,Z)-3,6-nonadien-1-ol], V24 (z-6-nonenal), V27 (3-carene), V34 (2-octyn-1-ol), V44 [Stearic acid, 3-(octadecyloxy) propyl ester], and V45 (10,12-Octadecadiynoic acid; Figure 2), and (E,Z)-3,6-Nonadien-1-ol was the representative volatile compound of CG considering the content (Table S1). V1 (ethyl acetate), V23 (hexyl acetate), and V20 (2-ethyl-2hexen-1-ol) were three principal contributors to PC1, when their abundance were taking into account (Figure 2, Table S1). We regarded ethyl acetate, hexyl acetate, (E, Z)-3, 6-nonadien-1-ol and 2-ethyl-2hexen-1-ol as four principal volatile compounds of these three types of melon. In Figure 3, it was obvious that acetate esters made “CH” or “CB” be separated from “CG.”

### Reductase activity of ADH and AAT activity in three types of melon at different DAP

**Figure 4:**
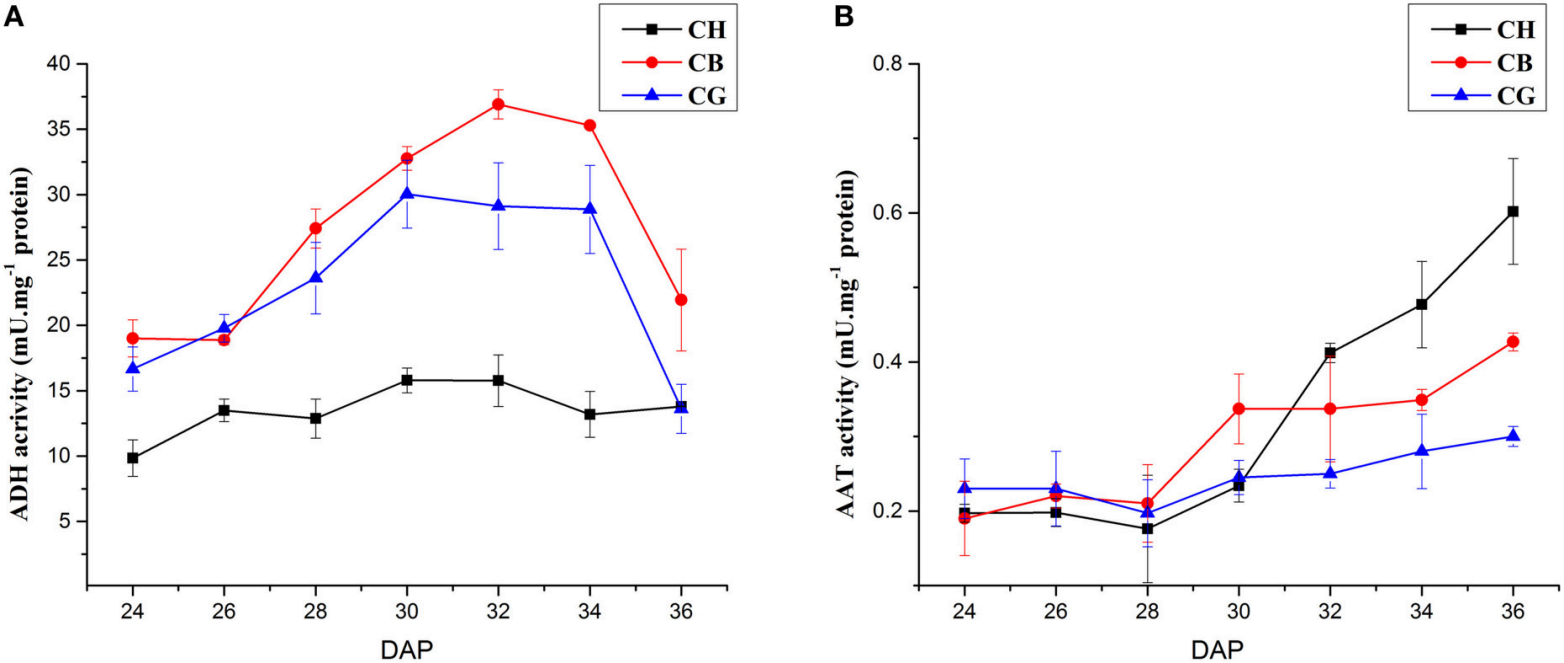
**ADH and AAT activities in three aroma types of melon at different DAP**. **(A)** ADH activities in three types of melon. **(B)** AAT activities in three types of melon. Each experiment was performed in triplicate and the means ± SE value of their activities were shown in the line chart.

During fruit development from 24 to 36 DAP, reductase activity of ADH in three types of melon showed a trend of increasing at first and decreasing subsequently, which reached a peak at 32 DAP. ADH activity was higher in flesh of CB and CG than that of CH from 24 to 36 DAP, but the change of ADH activity in flesh of CH was smaller than that of CB and CG (Figure 4A).

Figure 4B shows that AAT activity in flesh of CH significantly increased after 32 DAP and peaked on day 36. AAT activity in flesh of CB shows the similar change to CH, which increased after 30 DAP and peaked at 36 DAP, although the AAT activity in flesh of CB was lower than that of CH from 32 to 36 DAP. The AAT activity in CG did not change significantly and the level of enzyme activity in CG was the lowest among three melons from 32 to 36 DAP.

### *CmADHs* expression in three types of melon during fruit ripening

**Figure 5:**
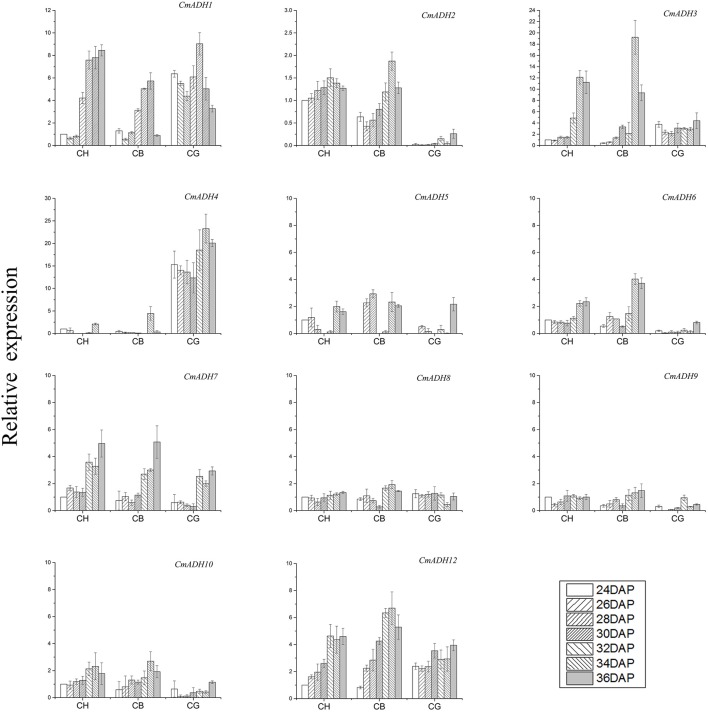
**Gene expression of ***CmADHs*** in three types of melon on different DAP**. Expression levels of each gene are showed as a ratio relative to the ADH/18SrRNA ratios for CH on 24DAP, which was set to 1. Each experiment was performed in triplicate and the means ± SE value of their content were shown in the figure.

A total of 11 *CmADHs* were expressed during ripening of melon (Figure 5), as *CmADH11* was not detected during our experiment. Transcript analysis indicate that these 11 CmADH genes were specifically expressed in ripening fruit of three aroma types of melon. *CmADH2* and *CmADH6* were specifically expressed in strong-aromatic melon CH and less-aromatic melon CB and *CmADH4* was only expressed in non-aromatic melon CG. The expression of *CmADH3, CmADH7*, and *CmADH12* in CH and CB were higher than that in CG, and most of the genes were consistently expressed with an increase in transcript abundance and reached the peak at 34 DAP or 36 DAP in CH and CB. In addition, the expression level of *CmADH5, CmADH8, CmADH9, CmADH10* were either not expressed or maintained a low level during fruit ripening.

### Volatile aroma compounds of CB in incubation experiment

**Figure 6:**
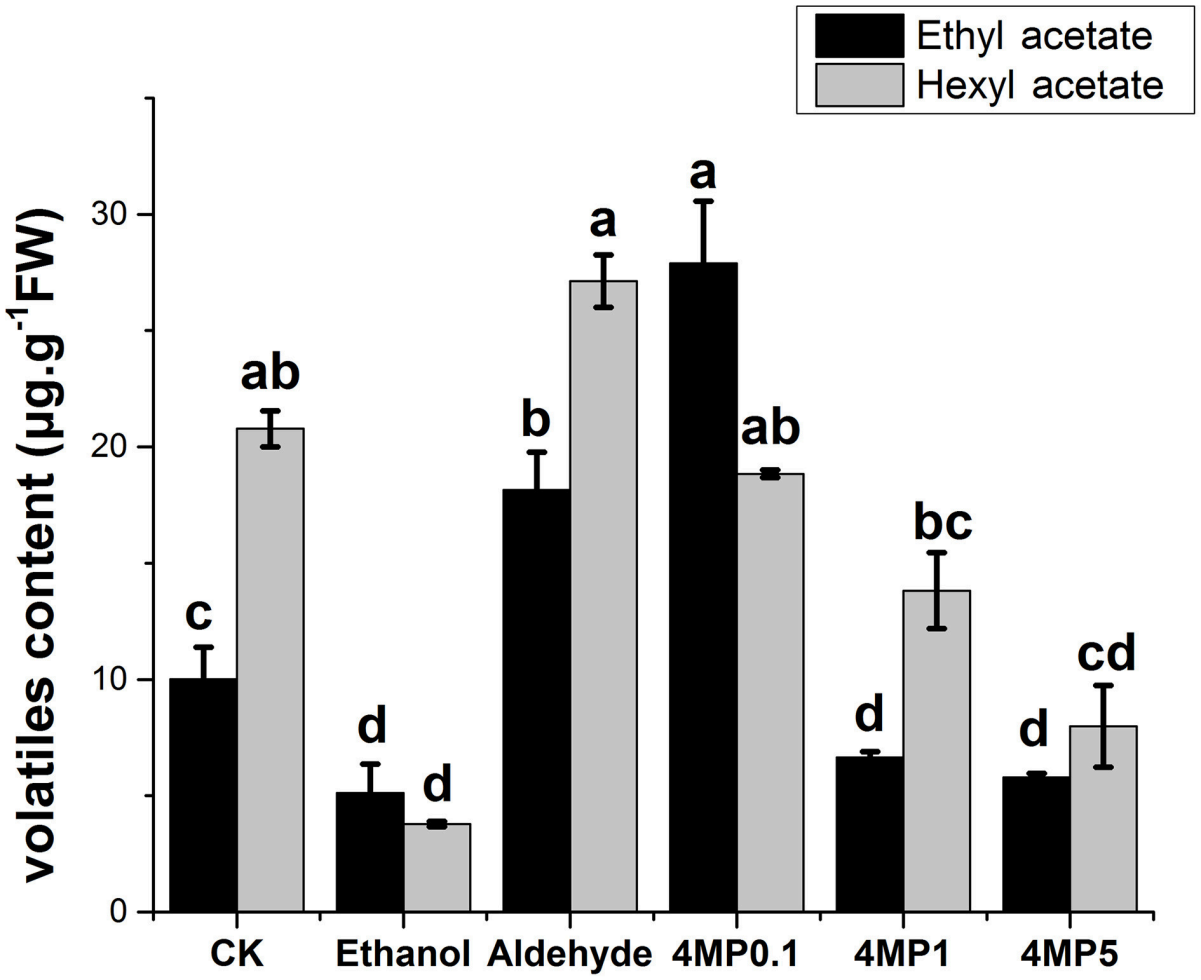
**Ethyl acetate and Hexyl acetate content in “CB” oriental melon in incubation experiment**. Flesh melon were incubated with 5 mM ethanol (Ethanol), 5 mM aldehyde (Aldehyde), 0.1 mM 4-methylpyrazole (4MP0.1), 1 mM 4-methylpyrazole (4MP1), and 5 mM 4-methylpyrazole (4MP5). Melon incubated with distilled water was taken as a control. Duncan's multiple range tests have been performed with different letters above the columns represent significant differences (*P* < 0.05) between different treatments.

Production of ethyl acetate and hexyl acetate in CB were significantly affected by substrates or inhibitor (Figure 6). Both ethyl acetate and hexyl acetate abundance reduced after ethanol treatment. Aldehyde only facilitated the production of ethyl acetate. The level of hexyl acetate was up-regulated, but it was not significant. For 4-methylpyrazole (4-MP), the inhibitor of ADH, it seems that the effect of 4-MP on melon acetate production was dose-dependent manner to some extent. Medium and high dose of 4-MP decreased the production of two acetates, while Low dose of 4-MP increased the ethyl acetate content (Figure 6).

### ADH activity in incubation experiment

**Figure 7:**
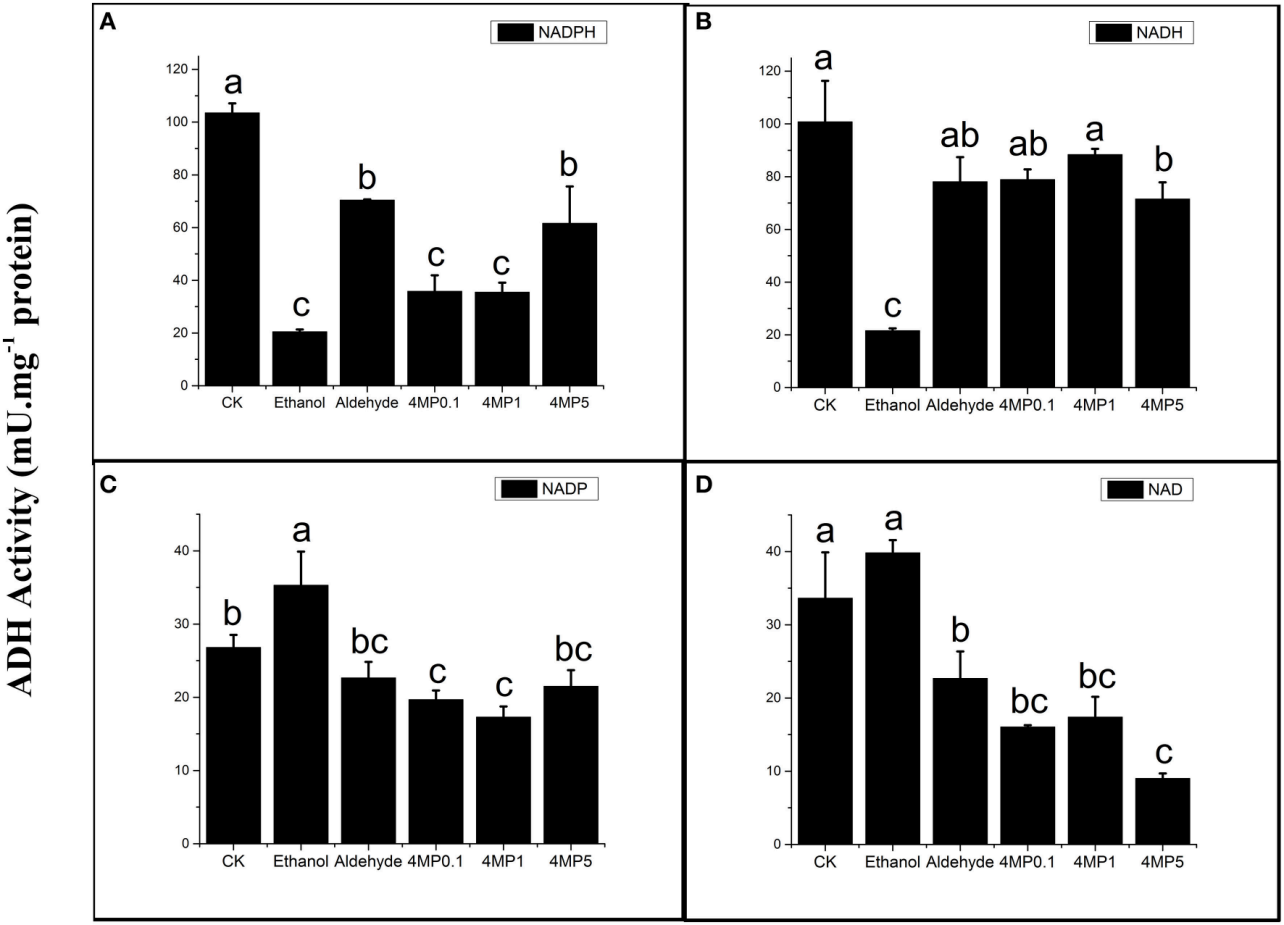
**ADH activities depended on four co-factors (0.25 mM NADPH/NADP and NADH/NAD) of CB flesh melon incubated with multiple solutions, including 5 mM ethanol (Ethanol), 5 mM aldehyde (Aldehyde), 0.1 mM 4-methylpyrazole (4MP0.1), 1 mM 4-methylpyrazole (4MP1), and 5 mM 4-methylpyrazole (4MP5) (A–D). (A)** ADH activity depended on 0.25 mM NADPH. **(B)** ADH activity depended on 0.25 mM NADH. **(C)** ADH activity depended on 0.25 mM NADP. **(D)** ADH activity depended on 0.25 mM NAD. Flesh melon incubated with distilled water were used as control. Duncan's multiple range tests have been performed with different letters above the columns represent significant differences (*P* < 0.05) between different treatments.

In incubation experiment, the ADH reductase activity was suppressed by ethanol, a production of ADH in melon, regardless of NADH or NADPH was used and ethanol showed a stronger suppression than 4-MP (Figures 7A,B). The dehydrogenase activity were increased by ethanol treatment, though activity change was more significant when the co-factor was NADP 4-MP also worked as an inhibitor, but it depended on co-factor and its concentration (Figures 7C,D). Aldehyde did not promoted the ADH reductase activity, but it significantly inhibited the dehydrogenase activity when the co-factor was NAD (Figure 7D).

### *CmADHs* expression in incubation experiment

**Figure 8:**
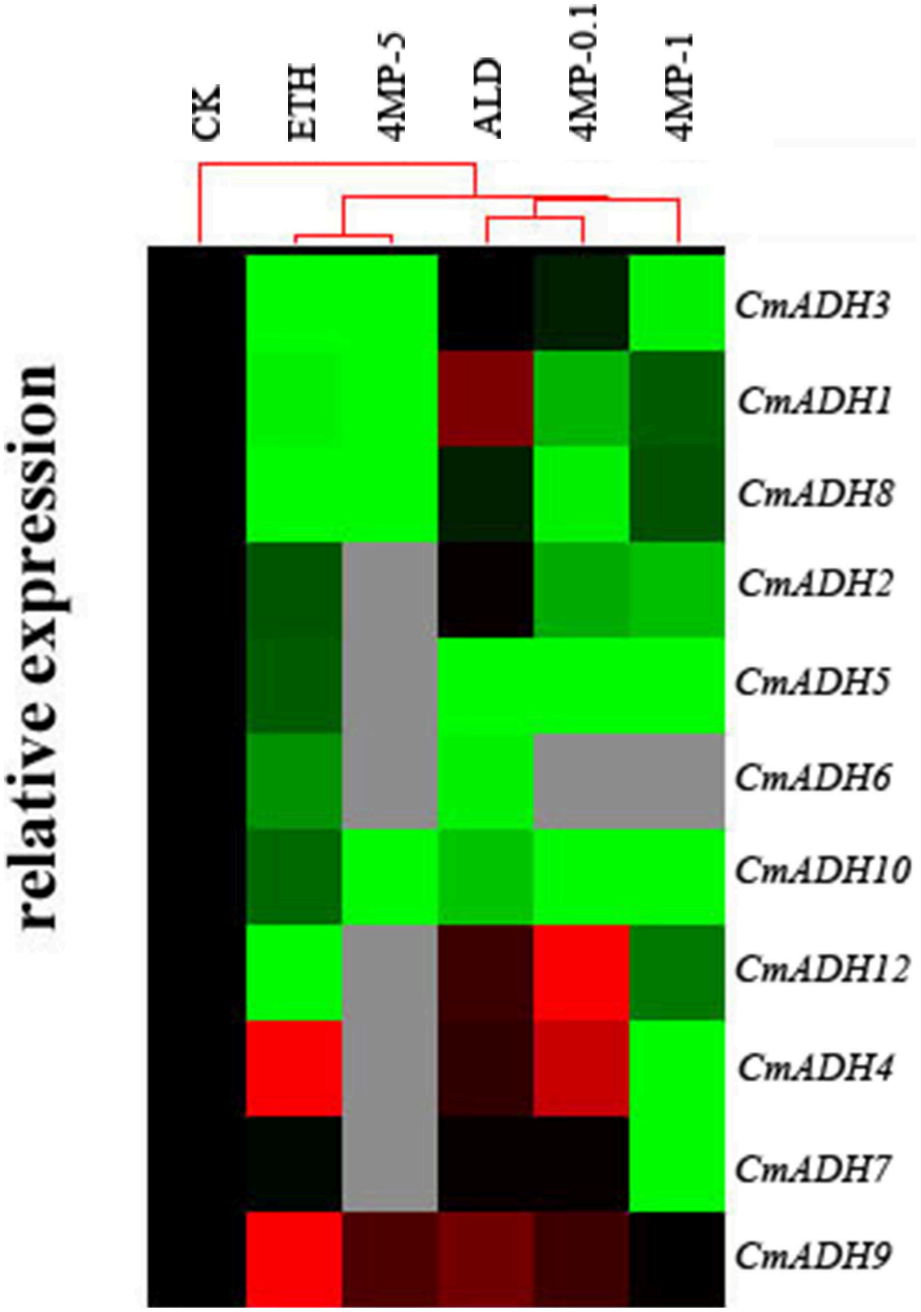
***CmADHs* gene expression in the incubation experiment of oriental melon “CB” flesh fruit**. Expression levels of each gene are showed as a ratio relative to the ADH/18SrRNA ratios for CK, which was set to black. The red cube means transcript level was up-regulated and the green cube means down-regulated on the contrary. All of the data for ADH gene expression are means of three replicates.

Based on the incubation experiment, 11 *CmADH* genes were expressed in oriental melon “CB” (Figure 8). *CmADH1, CmADH4, CmADH9*, and *CmADH12* were up-regulated following the addition of aldehyde, while *CmADH2, CmADH3, and CmADH7* seemed to not response to aldehyde. Most of *CmADHs* genes were down-regulated under ethanol treatment except *CmADH4, CmADH7*, and *CmADH9*. Different dose of 4-MP (0.1, 1, and 5 mM) reduced the levels of most *CmADHs* except *CmADH4, CmADH7, CmADH9, and CmADH12* (Figure 8).

## Discussion

Aroma was an important quality of ripe fruit, and it differed between varieties of the same species, which was found in many plants (Poll, 1981; Visai and Vanoli, 1997; Kourkoutas et al., 2006; Goulet et al., 2012). For example, esters and alcohols were the main aroma volatiles of Cantalope melon, sulfur esters and straight-chain compounds of six-carbon or nine-carbon were abundant, while E, Z-2,6-nonadienal was the principle aroma compound of honeydew melon fruit, and methyl esters were the main volatiles of Galia melon (Kourkoutas et al., 2006). Similar results were showed in high-aromatic melon Arava and less-aromatic melon Rochet; Acetate esters were abundant in Arava, while Rochet had high level of volatiles, such as alcohols and aldehydes (Shalit et al., 2001). In our study, unsurprisingly, except for soluble solids content and rind color, three types of melon showed diverse physiological characteristics in flavor, the aroma content of CH is the most abundant, either the total VOCs concentration or esters and CB content less esters than CH, but esters were still the most abundant volatile in CB flesh melon as well as CH; There were little esters in CG flesh melon, on the contrary, alcohols were the principle volatile of non-aromatic melon. Ethyl acetate and hexyl acetate were found to be the principle aroma compounds of CH and CB by PCA analysis combined with their content in ripening fruit, which was consistent with previous conclusion that volatile esters, especially straight-chain esters, were important VOCs in aromatic melon (Tang et al., 2015). In contrast, (E, Z)-3,6-nonadien-1-ol was the most abundant volatile in CG. These results illustrated that there were differences on the primary VOCs among different aroma types of melon and esters, especially ethyl esters were important aromatic compounds in oriental melons (Li et al., 2011; Liu et al., 2012).

The synthesis of straight-chain ethyl ester, such as hexyl acetate and butyl acetate, was directly correlated with the main enzymes activity in LOX pathway (Senesi et al., 2002; Echeverría et al., 2004; Altisent et al., 2009; Paige and Sheryl, 2012). ADH, as one of the key enzymes in LOX pathway, plays an important role in diverse volatile compounds synthesis in many plants. In olive, ADH activity may account for the diversity in aldehydes and alcohols of two cultivars, Carolea, and Coratina (Iaria et al., 2012). The high expression of *PuADH3* in pear during fruit ripening also indicated the relationship between *PuADHs* and aroma (Li et al., 2014). *CmADH1* and *CmADH2* were involved in fruit development due to their highly expression in Cantaloupe melon and ethylene-induced regulation. Particular substances preferences of two ADHs indicated their particular functions in the formation of various flavor of melon (Manríquez et al., 2006). But ADH is not the final step of LOX pathway, some alcohols produced by ADH would convert into esters under the function of AAT. So that there may be a complex relationship between ADH, AAT and volatiles: During the development of apricot fruit, the expression levels of *PaADH* and *PaLOX* stayed constant at all stages, however *PaAAT* levels showed a sharp increase in the late harvest stages, with the changes observed in ester levels (González-Agüero et al., 2009); Silencing *SlscADH1*, a specifically expressing gene in tomato fruit, resulted in the accumulation of C5 and C6 compounds rather than the alternation of alcohols/aldehydes balance (Moummou et al., 2012). In our study, ADH activity of all cultivars increased slightly first and raised up to several fold 2 days before the fruits ripened. There was no obvious difference between Less-aromatic melon CB with high esters content and non-aromatic CG with low esters content on ADH activity during fruit development, indicating that ADH activity might not be a key regulator of esters abundance in oriental melon. Increase of AAT activity was detected during ripening of fruit in CH and CB, but there was no significant change about AAT activity in non-aromatic melon CG. It seems there was no direct correlation between the total ADH activity and the total content of VOCs or the alcohols, and the AAT activity was positively correlated with the content of esters in oriental melons. The gene expression pattern of *CmADHs* also various in three cultivars during fruit ripening (Figure 5). The specific *CmADH* genes expression might be an important reason for the diversity of alcohols and follow-up ester components in oriental melon considering that different ADH had particular preferences for various substrates (Manríquez et al., 2006; Moummou et al., 2012) and further more studies are needed to prove the speculation.

We cannot analysis the specific substrate preference of each CmADH using crude enzyme, but the change of expression of every *CmADH* caused by some substrate could be detected in incubation experiment. Ethanol was immediate precursor of ethyl acetate, the most abundant characteristic aroma compound in oriental melon. Ethanol and aldehyde could be converted into each other by ADH through oxidation or reduction. Previous study showed that the exogenous application of ethanol could delay the maturation of oriental melon and increase the accumulation of aroma volatile compounds within a short time without influencing the ADH activity (Liu et al., 2012). Our study demonstrated ethanol significantly inhibited the activity of ADH enzyme of oriental fresh melon in incubation experiment, just like high concentration of 4-MP, the competitive inhibitor which could inhibit 40 to 60% of the *in vivo* activity of ADH in tomato (Beaulieu et al., 1997) or prevent the formation of ethanol (Kato-Noguchi and Yasuda, 2007), and the levels of most *CmADHs* expression were down-regulated with the reduction of esters. The confliction with former studies may be due to the concentration of ethanol treatment and the treatment time. Dehydrogenase activities of CmADH were slight deduced by aldehyde, but increase of reduction activities which we suspected were not found. The expression levels of *CmADH1, CmADH4, CmADH9*, and *CmADH12* in acetaldehyde treatment were improved, along with the production of ethyl acetate and hexyl acetate, suggesting their potential function in aroma volatile or ester synthesis. The results suggested that substrates were not the mainly regulator of *CmADHs* expression and ADH activity in oriental melon, and similar result was found in grapevine (Tesniere et al., 2004). Perhaps there was a complex regulation of *ADH* and enzymatic activity in oriental melon.

**Figure 9:**
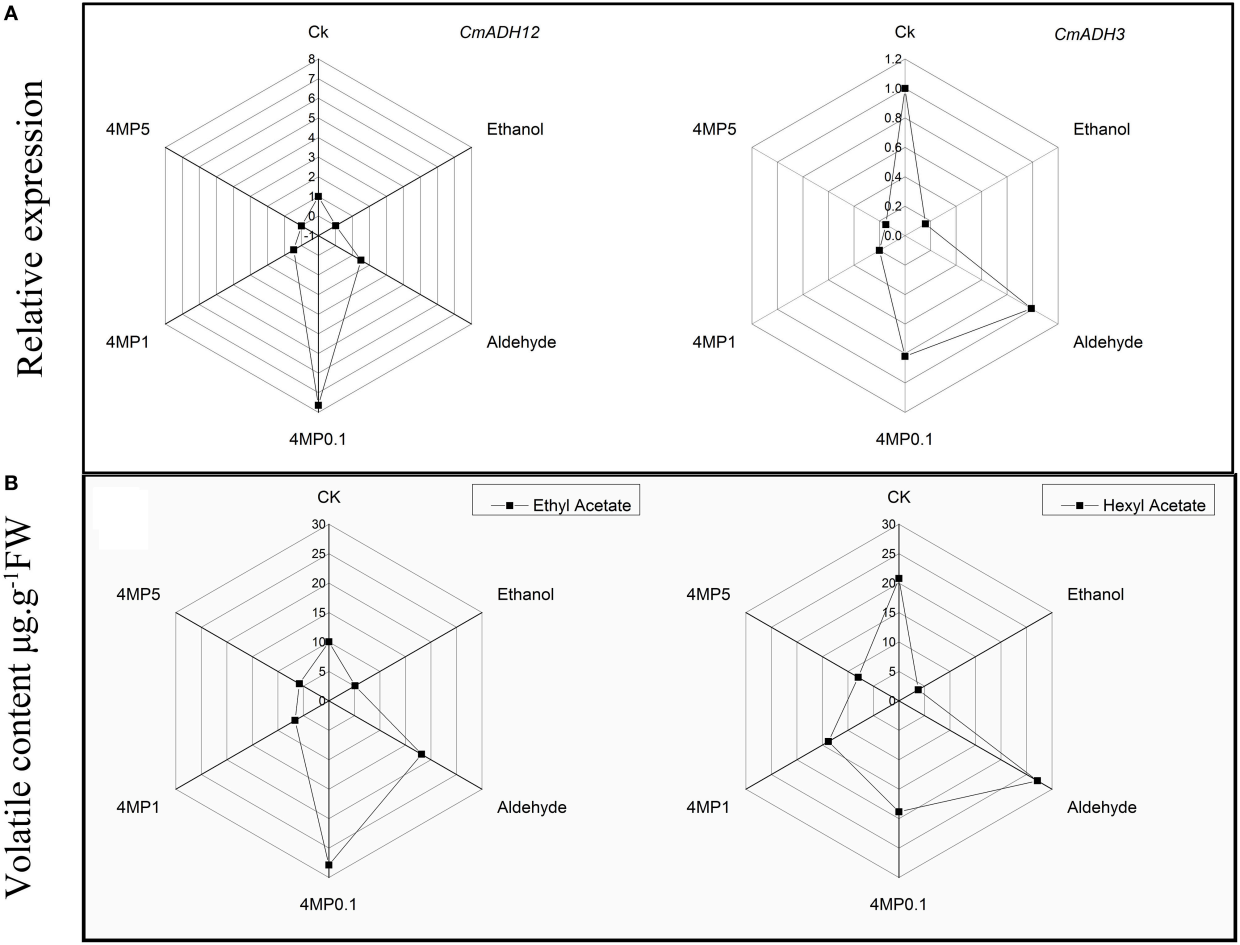
**Relative expression of two ***CmADH*** genes (***CmADH12*** and ***CmADH3***) and volatile content of two principle esters (ethyl acetate and hexyl acetate) in incubation experiment**. **(A)** *CmADH12* and *CmADH3* relative expressions in CB melon incubated with multiple treatments. **(B)** Volatile content of ethyl acetate and hexyl acetate in CB melon incubated with multiple treatments. The treatments were ethanol (Ethanol), aldehyde (Aldehyde), 0.1mM 4-MP (4MP0.1), 1mM 4-MP (4MP1), and 5mM 4-MP (4MP5). All of the data for ADH gene expression and volatile contents are means of three replicates.

So far, 12 CmAdh genes were found from the melon genome website and the function of most members were far from clear, except for *CmADH1* and *CmADH2* in Countloup melon. By bioinformatic analysis, we found that high homology appeared between *CmADH2* and *CmADH12* in spite of the low homology of the ADH gene family, and functional domains cheeked via NCBI's Conserved Domain Database suggested that CmADH12 might have the same catalytic function as short-chain dehydrogenases (Strommer, 2011; Jin et al., 2016). In addition, we were surprised to find the correlation among *CmADH3* or *CmADH12* gene expression pattern in our experiment, the ADH reductase activity when NADPH acted as the co-factor, and the accumulation of hexyl acetate or ethyl acetate in incubation experiment (Figure 9), although the *CmADH*s gene expression and the changes of enzyme activity would not directly affect the synthesis of esters in theory. It hinted *CmADH3* and *CmADH12* might involve in synthesis of aroma compounds of oriental melon. Considering that their expression levels were up-regulated by ethylene in our previous study (Jin et al., 2016). The recombinant protein or the transgenic plants were needed to obtain more information about the role of certain *CmADH*s in oriental melon aroma formation.

## Conclusions

In this paper, volatile esters, especially ethyl acetate, and hexyl acetate, as the primary aroma were identified in strong and less aromatic oriental melons, and alcohols, (E, Z)-3, 6-nonadien-1-ol, as the principle volatile, were also identified in non-aromatic melon. We found that the specific *CmADH* genes expression might be an important reason for the diversity of alcohols and follow-up ester components in three types of melon. ADH activity, *CmADH* genes expression and the content of two principle esters were significantly inhibited by ethanol, and the 4-MP, a kind of competitive inhibitor of ADH enzyme. While affection of aldehyde on *CmADH* activity or *CmADH* expression depended on co-factors or genes. We also found the relationship between *CmADH3, CmADH12* and the characteristic volatile, namely ethyl acetate or hexyl acetate. In conclusion, our study provide some evidences for the relationship between *CmADHs* and volatile compounds of oriental melon, and more studies are needed to make it clear.

## Author contributions

HC and HQ designed research; HC performed research; HC analyzed data; HC and HQ wrote the paper; SC, YJ, and YT helped to revise the paper.

## Funding

First level of Liaoning high school Talent support program, LR2014020.

### Conflict of interest statement

The authors declare that the research was conducted in the absence of any commercial or financial relationships that could be construed as a potential conflict of interest.

## Supplementary material

Table S1 **Volatile compounds and their concentrations (μg.g-1FW) in different aroma types of the melon ripen fruit. Include “Cai Hong” (CH), “Cui Bao” (CB) and “Cai Gua” (CG)**. Each experiment was performed in triplicate and the mean value of their concentrations were shown in this table.

Figure S1 **The SSC of three types of melon at different days after pollination (DAP)**. The three aroma types melon are CH (short for “Cai Hong”), CB (short for “Cui Bao”), and CG (short for “Cai Gua”). Each experiment was performed in triplicate and the means ± SE value of their content were shown in the line chart.

Figure S2 **Different appearance of three types of melon (***Cucumis melo***). (A)** Oriental melon (*C. melo* var. *makuwa* Makino) cultivar “Chai Hong” (CH). **(B)** Oriental melon (*C. melo* var*. makuwa* Makino) cultivar “Chai Hong” (CH). **(B)** Oriental melon (*C. melo* var*. makuwa* Makino) cultivar “Cui Bao” (CB). **(C)** Snake melon (*C. melo* L. var*. flexuosus* Naud) “Cai Gua” (CG).
