# Supplementary material for: The Relationship between CmADHs and the Diversity of Volatile Organic Compounds of Three Aroma Types of Melon (Cucumis melo)
Source: Front Physiol. 2016 Jun 28;7:254. doi: 10.3389/fphys.2016.00254 (PMC4923263; doi:10.3389/fphys.2016.00254)
Supplement: Table S1 — Volatile compounds and their concentrations (μg.g-1FW) in different aroma types of the melon ripen fruit. Include “Cai Hong” (CH), “Cui Bao” (CB) and “Cai Gua” (CG). Each experiment was performed in triplicate and the mean value of their concentrations were shown in this table. [file TableS1.DOC]

| **S1 Table** | | | | |
| --- | --- | --- | --- | --- |
| Volatile compounds  (μg.g-1FW) | Cas | Different types of melon | | |
|  |  | CH | CB | CG |
| V1 Ethyl Acetate | 141-78-6 | 86.29 | 58.94 | 2.55 |
| V2 3-Pentanethiol | 616-31-9 | 0.20 | ND | ND |
| V3 1,3-Dimercaptopropane | 109-80-8 | ND | 0.27 | ND |
| V4 Propanoic acid | 79-09-4 | 3.09 | 3.70 | ND |
| V5 Acetic acid | 64-19-7 | 18.22 | ND | ND |
| V6 6-Nonynoic acid | 56630-31-0 | ND | 1.70 | ND |
| V7 Propanoic acid, 2-methyl- | 79-31-2 | 2.06 | 1.61 | ND |
| V8 Butanoic acid, 2-methyl-, methyl ester | 868-57-5 | 3.17 | ND | ND |
| V9 Hexadecanoic acid, ethyl ester | 628-97-7 | ND | 2.75 | ND |
| V10 Ethyl 4-bromobutyrate | 2969-81-5 | 13.02 | 10.19 | 0.97 |
| V11 3,6-Octadecadiynoic acid, methyl ester | 56554-43-9 | ND | 0.74 | ND |
| V12 Butanoic acid, 2-methyl-, ethyl ester | 7452-79-1 | 8.75 | 9.08 | 0.55 |
| V13 2-Hexenal, 2-methyl- | 5187-71-3 | ND | 1.02 | ND |
| V14 1-Butanol, 2-methyl-, acetate | 624-41-9 | 7.90 | ND | 0.64 |
| V15 5-Hexen-2-ol, 5-methyl- | 50551-88-7 | 1.00 | 0.96 | 0.55 |
| V16 Thiopivalic acid | 55561-02-9 | 4.46 | 0.89 | ND |
| V17 Acetic acid, pentyl ester | 628-63-7 | ND | 0.33 | ND |
| V18 2-Heptyn-1-ol | 1002-36-4 | ND | 6.53 | ND |
| V19 1-Pentanol | 71-41-0 | 1.72 | 1.21 |  |
| V20 2-ethyl-2-Hexen-1-ol | ND | 4.59 | 8.56 | 1.02 |
| V21 Ethyl (methylthio)acetate | 4455-13-4 | 5.86 | ND | ND |
| V22 2-Hexadecanol | 14852-31-4 | 0.18 | ND | ND |
| V23 Hexyl acetate | 142-92-7 | 12.98 | 13.12 | ND |
| V24 6-Nonenal, (Z)- | 2277-19-2 | ND | ND | 4.49 |
| V25 2-Octenal, (E)- | 2548-87-0 | 0.39 | 0.97 | 0.16 |
| V26 Benzeneacetaldehyde | 122-78-1 | 2.93 | ND | ND |
| V27 3-Carene | 13466-78-9 | ND | ND | 0.64 |
| V28 2,3-Butanedioldiacetate | 1114-92-7 | 9.86 | ND | ND |
| V29 Trans,cis-3,6-nonadien-1-ol | 56805-23-3 | 14.58 | ND | 50.10 |
| V30 Octen-1-ol, acetate | 32717-31-0 | 8.76 | 4.80 | ND |
| V31 Stearic acid, 3-(octadecyloxy)propyl ester | 17367-40-7 | 1.36 | ND | ND |
| V32 2,3-Epoxycarane, (E)- | 20053-58-1 | ND | 0.05 | ND |
| V33 Acetic acid, phenylmethyl ester | 140-11-4 | 1.78 | 2.27 | ND |
| V34 2-Octyn-1-ol | 20739-58-6 | 5.91 | ND | 16.89 |
| V35 9-Octadecen-12-ynoic acid, methyl ester | 56847-05-3 | 3.64 | 1.39 | ND |
| V36 9,12-Octadecadienoic acid (Z,Z)- | 60-33-3 | 2.92 | ND | ND |
| V37 9-Tetradecen-1-ol, acetate, (E)- | 23192-82-7 | 3.66 | 1.58 | ND |
| V38 Propanoic acid, 2-methyl-, ethyl ester | 97-62-1 | 6.76 | 1.29 | ND |
| V39 1-Dodecanol, 3,7,11-trimethyl- | 6750-34-1 | 3.18 | ND | ND |
| V40 2,5-Octadecadiynoic acid, methyl ester | 57156-91-9 | 14.57 | 5.27 | ND |
| V41 Ionone | 8013-90-9 | 11.24 | ND | ND |
| V42 Eugenol | 97-53-0 | 5.80 | 4.12 | ND |
| V43 5,8,11-Heptadecatriynoic acid, methyl ester | 56554-57-5 | 10.77 | ND | ND |
| V44 Stearic acid, 3-(octadecyloxy)propyl ester | 17367-40-7 | ND | ND | 1.86 |
| V45 10,12-Octadecadiynoic acid | 7333-25-7 | ND | ND | 0.63 |
| V46 Farnesene | 502-61-4 | 23.90 | 6.48 | ND |
| V47 Phenol, 2,4-bis(1,1-dimethylethyl)- | 96-76-4 | ND | ND | 61.46 |
| V48 Cedrol | 77-53-2 | ND | ND | 11.68 |
| V49 Decanoic acid, decyl ester | 1654-86-0 | 6.39 | 3.29 | ND |

**Note: ND, not be detected.**

**Table 1. Total and different classes of volatile compounds and their concentrations in different aromatic melon types.**

| Volatile compounds (μg.g^-1^ FW) | Different types of melon | | |
| --- | --- | --- | --- |
|  | CH | CB | CG |
| Total esters | 207.83±17.21a | 127.16±16.75b | 5.29±1.82c |
| Total alcohols | 30.24±2.48b | 23.68±1.87b | 136.85±4.85a |
| Total acids | 15.73±5.28a | 8.14±2.62b | 9.31±3.97b |
| Others | 54.15±20.01a | 10.07±1.32b | 6.14±1.21b |
| Total aroma | 307.96±5.59a | 169.05±22.56b | 144.60±13.56b |
