# Supplementary figures and images for: The Relationship between CmADHs and the Diversity of Volatile Organic Compounds of Three Aroma Types of Melon (Cucumis melo)
Source: Front Physiol. 2016 Jun 28;7:254. doi: 10.3389/fphys.2016.00254 (PMC4923263; doi:10.3389/fphys.2016.00254)

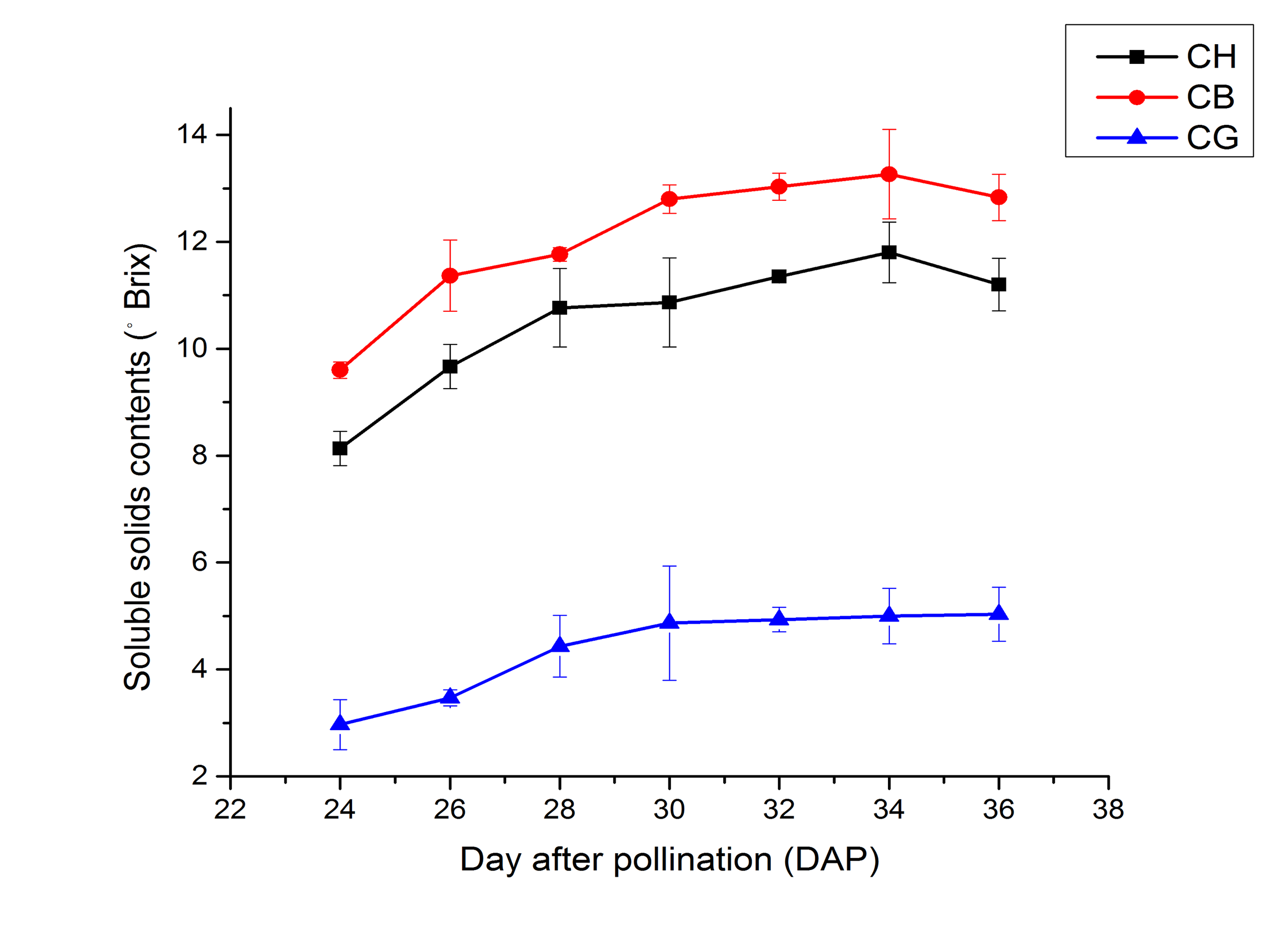

Supplement: Figure S1 — The SSC of three types of melon at different days after pollination (DAP). The three aroma types melon are CH (short for “Cai Hong”), CB (short for “Cui Bao”), and CG (short for “Cai Gua”). Each experiment was performed in triplicate and the means ± SE value of their content were shown in the line chart. [file Image1.tif]

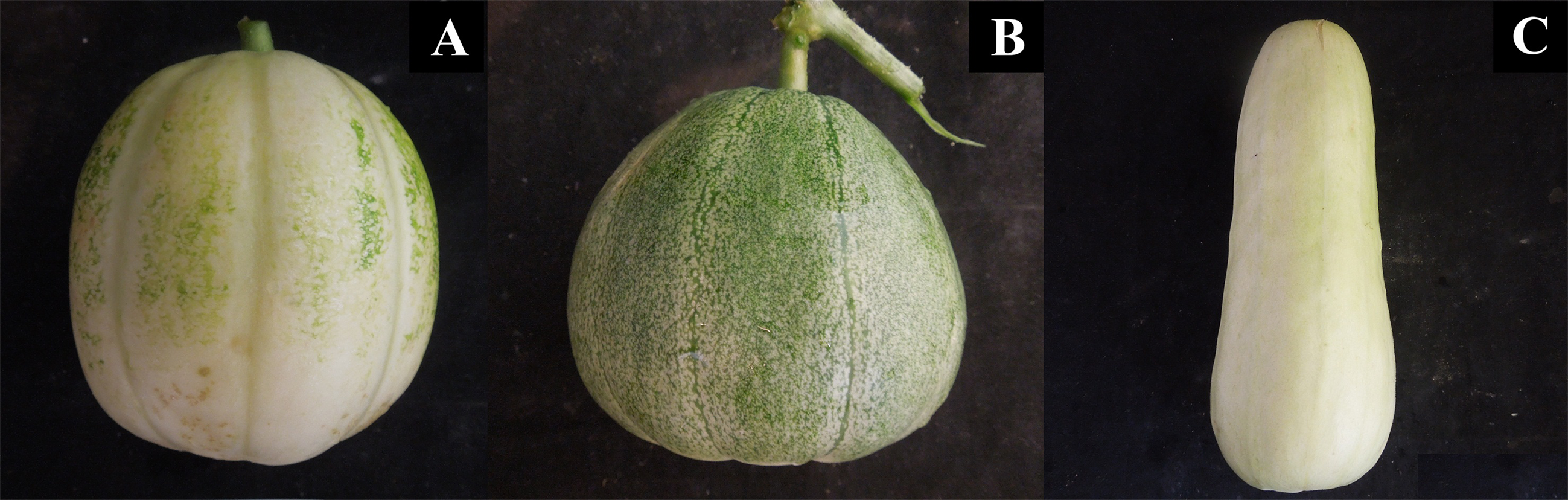

Supplement: Figure S2 — Different appearance of three types of melon (Cucumis melo). (A) Oriental melon (C. melo var. makuwa Makino) cultivar “Chai Hong” (CH). (B) Oriental melon (C. melo var. makuwa Makino) cultivar “Chai Hong” (CH). (B) Oriental melon (C. melo var. makuwa Makino) cultivar “Cui Bao” (CB). (C) Snake melon (C. melo L. var. flexuosus Naud) “Cai Gua” (CG). [file Image2.tif]
